# Innovating Challenges and Experiences in Emory Health AI Bias Datathon: Experience Report

**DOI:** 10.1007/s10278-024-01367-5

**Published:** 2025-02-25

**Authors:** Atika Rahman Paddo, Saptarshi Purkayastha, Janice Newsome, Hari Trivedi, Judy Wawira Gichoya

**Affiliations:** 1 Department of BioHealth Informatics, Indiana University Indianapolis, 535 W Michigan St, Indianapolis, 46202 IN USA; 2 Department of Radiology, Emory University School of Medicine, 100 Woodruff Circle, Atlanta, 30322 GA USA

**Keywords:** Datathon, Healthcare innovation, Participant experiences, Post-event survey

## Abstract

This paper presents an in-depth analysis of the Emory Health AI (Artificial Intelligence) Bias Datathon held in August 2023, providing insights into the experiences gained during the event. The datathon, focusing on health-related issues, attracted diverse participants, including professionals, researchers, and students from various backgrounds. The paper discusses the preparation, organization, and execution of the datathon, detailing the registration process, team formulation, dataset creation, and logistical aspects. We also explore the achievements and personal experiences of participants, highlighting their resilience, dedication, and innovative contributions. The findings include a breakdown of participant demographics, responses to post-event surveys, and participant backgrounds. Observing the trends, we believe the lessons learned, and the overall impact of the Emory Health AI Bias Datathon on the participants and the field of health data science will contribute significantly in organizing future datathons.

## Introduction

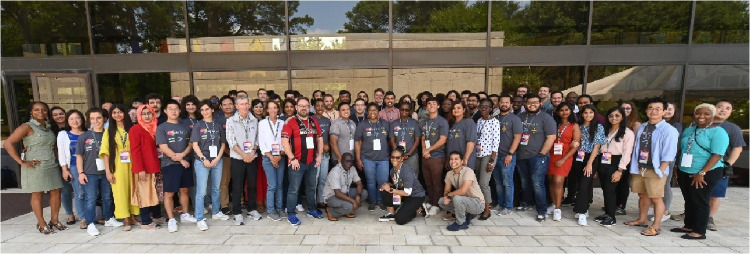



Participation in conferences or research symposiums constitutes a valuable element in the educational and professional development of students and researchers [1]. Such events facilitate meaningful interactions with peers and exposes attendees to novel research, thereby fostering the generation of innovative ideas. Similarly, the impact of events like datathons (or Hackathons) is emerging as particularly profound, as they catalyze collaborations between individuals from diverse sectors to develop novel solutions to complex challenges [2, 3]. Health datathons, in particular, promote diversity in collaboration in medicine [4], and help in bringing real-world health challenges to newer researchers [5, 6]. Trying to achieve meaningful insights from solving datathon challenges also facilitates addressing common challenges in healthcare industries as the collaborative nature of health datathons enables individuals with diverse backgrounds and varying expertise in the health sector to join forces in devising innovative solutions for the presented health-related problems.

The Emory Health AI Bias Datathon conducted from August 17 to 21, 2023, exemplified the trend toward collaborative applied AI research by bringing together a diverse group to address pressing issues in healthcare AI. An analysis of datathon registration, activities, and post-event surveys yielded rich insights. These findings can inform best practices for planning inclusive interdisciplinary datathons focused on mitigating AI bias in healthcare and beyond. The datathon formulated unique health-focused AI challenges and convened an interdisciplinary cohort of researchers, professionals, and students. Their varied backgrounds enabled multifaceted perspectives. Participant data revealed demographic trends, experiential insights, and recommendations that future organizers can use to enhance datathon impact. This case study demonstrates the value of collaborative AI ethics inquiry [7]. It provides a model for orchestrating productive interdisciplinary datathons to advance equitable and transparent AI. After the datathon event, we also collected feedback and responses from several participants in a systematic format which will guide the organizers of future events.

## Background

Health hackathons or datathons bring together individuals from diverse backgrounds, including healthcare professionals, data scientists, programmers, designers, and entrepreneurs, to collaboratively work on solving challenges related to healthcare using data-driven approaches over a short period of time, usually a day or two. These hackathons aim to leverage the power of data and technology to find innovative solutions, improve patient outcomes, and enhance the overall healthcare ecosystem [8, 9]. On most occasions, datathons are focused on tackling specific healthcare challenges or questions. The solutions and insights generated during datathons can inform new research directions, products, and services that improve clinical care, public health, patient engagement, and more. For example, Israel’s Ministry of Health (MoH) held a virtual datathon, and Israel’s research community was invited to offer insights from the datathon to help solve COVID-19 policy challenges [10]. Based on participants’ feedback from the datathon, the future data-driven regulatory response process for health crises was improved. Also, a research team at the 2018 Singapore healthcare datathon had identified a potential optimal SpO2 range for critically ill patients requiring oxygen therapy [11]. As the datathons often direct their challenges to specific problems, they often make large datasets available from healthcare systems, research studies, or other sources, which makes it easier for researchers to use. Ideas such as how transfer learning can be simplified to develop predictive models for small chest X-ray datasets [12] often can be generated by experts while dealing with such datasets in a datathon-like environment. Nicholas et al. [13] discuss educational applications of Health Gym’s synthetic datasets used in a health datathon where research teams developed and evaluated a transformed MIMIC database to OMOP over a 48-h long health datathon [14]. Long before MIT and People’s Liberation Army General Hospital held China’s first health datathon, fostering collaboration among clinicians, statisticians, and data scientists to address ICU information gaps using electronic health records (EHRs) [15]. Serpa et al. [16] also reported the first datathon on critical care held in Brazil. A study by de et al. [17] showed that healthcare datathons assemble diverse participants to collaboratively conduct clinically relevant research or develop algorithms, traditionally evaluated by criteria such as publications, patents, and startup companies, from their study of 50 participants revealing that these events enhance affective learning and teamwork, highlighting the crucial role of effective leadership. Also, healthcare datathons in Sydney, Singapore, and Beijing, with a 64% response rate (301/467) from predominantly male participants, health professionals, and data scientists, indicate positive learning experiences, content satisfaction, behavioral application, mentor instruction, and future participation intent, highlighting datathons’ efficacy in promoting cross-disciplinary collaboration, healthcare improvement through data science, and big data analytics preparation [18].

Healthcare digitization and a persistent gap between clinicians and data scientists leads to unresolved quality issues. According to Celi et al. [19], bridging this gap through collaborative events like datathons is crucial, but a more significant transformation in medical education and funding processes is needed for a successful learning health care system. To organize a successful datathon, Sobel et al. [20] provided a checklist of venue format, logistics, partnerships, well-defined projects, IT support, experienced mentors, targeted participants, effective communication, and awards to support project continuation. Aboab et al. [21] also proposed a “datathon” or “hackathon” model to encourage collaborative efforts among participants with diverse knowledge and skills to address clinical questions, promoting continuous peer review through follow-up datathons for more robust and reliable research outcomes.

## The Datathon Process

A large team of experts, data scientists, and professionals worked toward building the infrastructure, creating and processing the datasets to be used in the datathon, and creating documentation and tutorials. Parts of their tasks included formulating teams, creating the Emory ICU and chest X-ray dataset from scratch using data from several hospitals under Emory Healthcare, checking and preparing the logistics, and inviting speakers from different parts of the world.

### Preparation

The datathon organizers began preparations nearly 2 months before the event, forming teams of experts with diverse backgrounds. These teams worked collaboratively to build servers tailored for different participant groups in the datathon. The participant teams, each assigned specific datasets, servers, and project statements, focused on diverse topics such as validating the ALP score for osteoarthritis assessment, detecting and mitigating bias in medical imaging algorithms, evaluating biases in the Emory ICU dataset, and testing the performance of large language models, particularly ChatGPT for health.

### Registration

A total of 112 participants registered for the datathon event. While many registered for the 3rd-day symposium as well in the event. Several groups were created based on the registration information provided to the participants, ensembling participants from different backgrounds and professions. The participant teams were also formed based on responses gathered during the registration process, where participants shared information about their achievements, failures, advice, fun facts, and inspiration. Organizers categorized these responses as positive, negative, or neutral. Among the participants, 29.2% of the people showed positive responses, 20.2% showed negative responses, and 50.6% showed neutral responses. Teams were then strategically composed to include individuals with a mix of positive, negative, and neutral overall responses, fostering a diverse and balanced collaborative environment within each team. The datathon also registered 17 mentors for the teams; three of them mentored all teams throughout the event days.

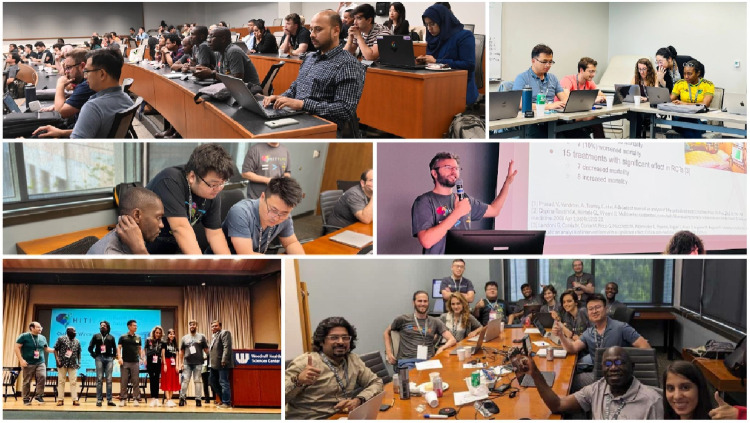



### Day 1

The event opening program started on Day 1. On-site registration was available for the participants throughout the day. The event started with a reception of the participants, and a cultural performance was conducted by Uhuru Dancers from Atlanta. Simultaneously, a networking event among the participants was conducted.

### Day 2

The organizers briefed everyone about the datathon datasets and challenges at the beginning of Day 2, after which the datathon started. The datasets for the specific problem statements included the EMory BrEast imaging Dataset (EMBED) [22], the Emory Knee Radiography Dataset (MRKR), Emory chest X-ray and ICU dataset. In total, 13 groups were formulated before that. Each participant was also given the opportunity to choose their dataset and change the groups if they wanted.

### Day 3

The datathon continued on Day 3, and each group submitted their final findings at mid-noon. At the end of the day, all groups presented their work through separate presentations.

### Day 4

The day started with the inauguration of the symposium that was planned to be held in conjunction with the main datathon event. The symposium ran simultaneously while researchers and professionals held discussions with panel members, alongside question-answer sessions. At the end of the symposium, winners from the datathon were announced and prized.

### Post-Event Survey

Significant numbers of participants from the datathon also participated in a post-datathon survey regarding their experiences from the event. This post-event survey was distributed through email, and their consent was collected through the online questionnaire. The questionnaire was prepared according to their personal and professional experiences and overall datathon participation.

## Findings

### Demographics

**Fig. 1:**
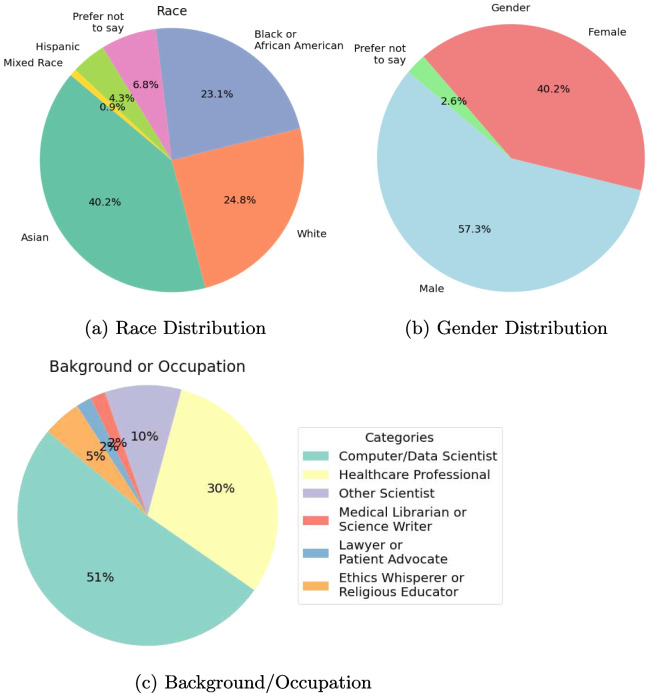
Participant demographics

The demographic distribution of participants in the datathon is visually depicted in Fig. 1a and b. Analysis reveals a predominant representation of Asian individuals, and the overall participant pool demonstrates a slightly higher ratio of male participants.

The background of the participants in the datathon is shown in Fig. 1c. It is observed that mostly computer/data scientists participated in the datathon.

**Fig. 2:**
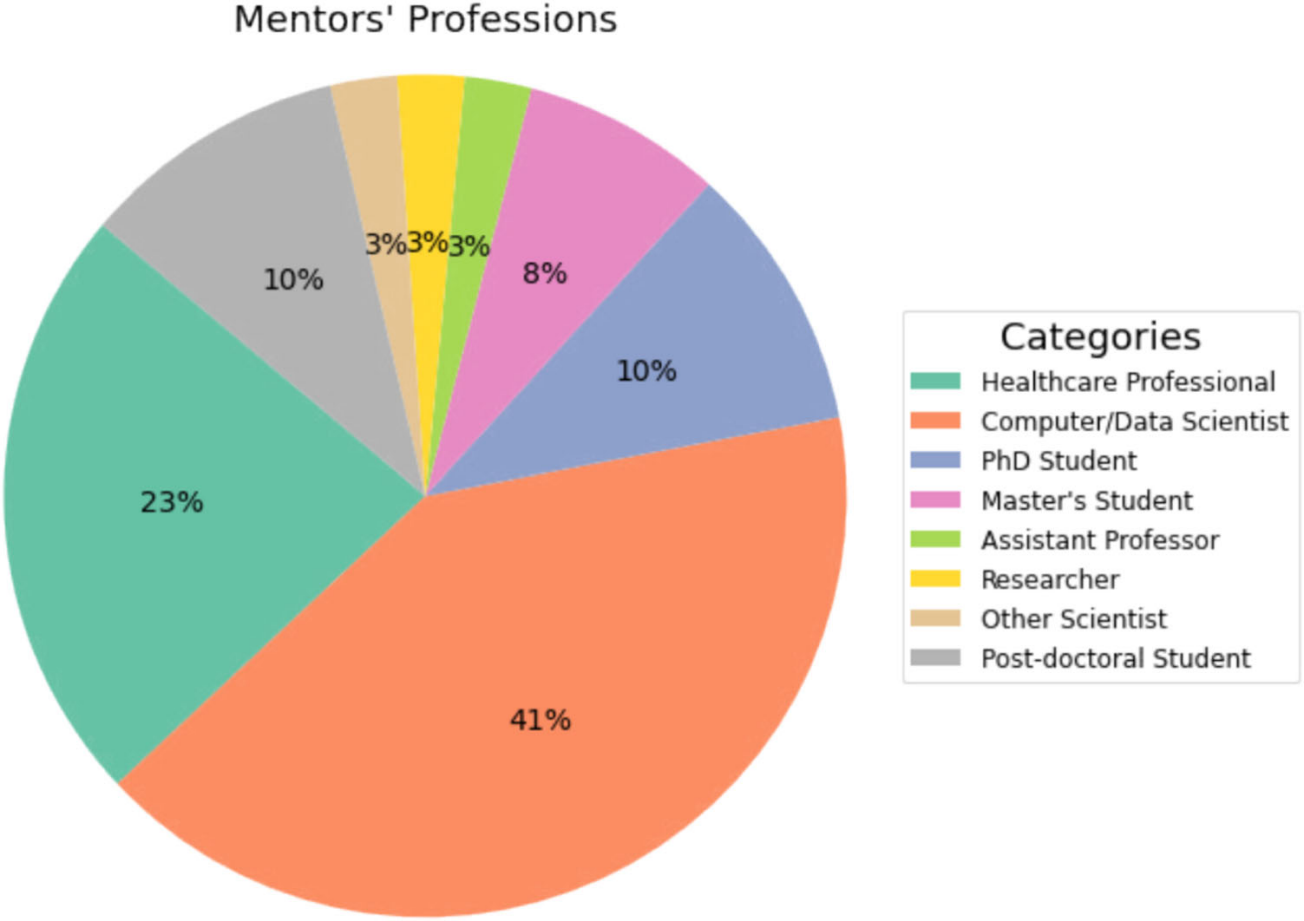
Distribution of mentors’ profession

### Responses from the Participants

#### Achievements

We asked the participants during registration of their personal achievements that deserve recognition and the accomplishments that they take great pride in, the challenges they overcame to attain, and valuable lessons they learned from the experiences, hoping to that their experiences would make the participants connect to each other and get a better network opportunity. The responses from the participants highlight a diverse range of personal achievements, each accompanied by unique challenges and valuable lessons. One participant shared their dedication to achieving “top talent” in national piano guild auditions, emphasizing the importance of intentional practice, putting in the reps, and staying focused. They say, “I practiced multiple hours a day for weeks to the point that I started getting hand pain/bumps on my wrists - it taught me the importance of intentional practice, the importance of putting in the reps and staying focused.”

Another participant narrates their journey of pursuing a Ph.D. as a single mother, overcoming classmate criticism and societal expectations, and ultimately becoming a source of inspiration for their daughter, now in medical school. They state, “My daughter is now in medical school, and she points to me for her reason to believe that she can face down anything. She was taking her resilience cues from me all along, so I’m happy I made it through!” Another participant shared about their evolving medical career, winning prizes for innovative projects to address health challenges in low- and middle-income countries in Africa.

Recognition and awards play a significant role, as one participant having been two prestigious awards emphasizes, “Essentially both these awards are special to me because they essentially come from the field people who knew me till my PhD would be surprised to see I work in the field I have been awarded the recognition.” Completing a Ph.D. and being the first in the extended family, building real-time dashboards during the COVID-19 pandemic, and winning best poster awards in emerging fields showcase adaptability and a willingness to explore new domains. One participant reflects, “Nothing can stop you from doing anything if you have the right energy, enthusiasm, and strength to get it done.”

Another participant reflects on their growth from struggling with coding during a move to the USA for a master’s program to co-authoring research papers and contributing to their field. They state, “This is something I believe is real growth and achievement.” Several respondents shared data science and health informatics achievements, from predicting brain strokes to managing large datasets for a global public health project. Personal challenges, such as being a first-generation student accepted into a Ph.D. program, learning to code within a week for a research project, and balancing family responsibilities while pursuing academic and professional goals, add a human touch to the narratives. One participant highlights the significance of being a mother and a respiratory therapist, underscoring the importance of prioritizing family amidst professional pursuits. They share, “Moments will never be repeated; seek to enjoy the present moment.”

The stories also encompass a diverse range of accomplishments, from opening an art market and coffee shop during the pandemic to successfully co-founding and chairing a health datathon. The multidisciplinary nature of achievements is evident, from teaching and art to patient safety and gig economy entrepreneurship. One participant expressed, “I learned a great deal in the last three years, and I stay curious about what life will continue to bring us as we lean into new ways of being and flourishing.”

#### Failure

We also asked the participants about their failures in life, specifically how they managed to overcome failure and the valuable lessons they learned from the experience. They shared a variety of instances where they experienced failure and the lessons learned from those experiences. One respondent stated, “Took 9 years to get my black belt in taekwondo (average is  3 years), stubborn persistence and continuing to show up, be faithful in the process - outcomes will come.” Another participant emphasized the challenge of obtaining data for research, noting, “The main lesson was to keep pushing.” In the realm of academia, a person faced the possibility of leaving academia but, after a failed job application, took it as a sign to stay in the academic field, stating, “I always see failure as an opportunity to change my process.”

A respondent acknowledged the unpredictability of outcomes for those involved in technology innovations, saying, “Lessons learned - always expect things to be different than you planned.” Another participant, an Innovations Manager, shared a valuable lesson, “Always express your challenges. It becomes easier to get the right help when people know what you are facing.”

A respondent who faced visa denials for post-graduate studies in the USA shared, “I started to think I would never be able to pursue post-graduate studies. However, after an initial setback of 3–4 months, I started looking for opportunities outside the states, which allowed me to pursue unknown pastures in Australia.” The emphasis on persistence, self-belief, and adaptability were recurring themes in these narratives.

In the field of education, failures in admission exams and rejection from medical courses were turned into opportunities for personal growth. One participant noted, “I accepted the course that was offered to me and focused to excel in it.” Facing rejection in the job market, another participant’s proactive approach included, “The lesson I learned is that all fields are good as long as one works hard to be among the best.”

An internship seeker faced multiple rejections but, by appealing a rejection, managed to turn the tide and secure a valuable opportunity, stating, “This experience taught me several valuable lessons.” In the field of health informatics, a setback in harmonizing data during an internship was seen as an opportunity for growth, with the respondent noting, “This experience was a watershed moment for me, teaching me that failure is not an insurmountable barrier but rather a catalyst for learning and growth.”

Failures in balancing commitments during college underscored the importance of understanding one’s limits and prioritizing effectively, with one participant sharing, “It was a harsh lesson in understanding my limits.” Respondents shared personal experiences from various fields, including academia, technology, healthcare, and personal development, using direct quotations to convey their perspectives on resilience and lessons learned from navigating challenges and setbacks (Fig. 2).

#### Inspirations

We asked the participants regarding who or what inspired them to be in their fields. They shared their unique paths to their respective fields, each driven by distinct sources of inspiration. One respondent discovered their passion for information through a reference and bibliography course during their undergraduate studies, stating, “I had the opportunity to take a reference and bibliography course and fell in love with the process of information. That one course changed the trajectory of my career goals.” Another individual, inspired by technology’s potential to enhance lives, credited specific mentors, saying, “They gave me the inspiration and an opportunity. I’ll forever be grateful for that.” In the realm of medical and healthcare fields, motivations were deeply personal. An ophthalmologist eloquently expressed their admiration for the eye, noting, “The eye is a ‘holy’ place, like a holy, revered temple. It is so precious, so elegant in its design and structure, so detailed, so precise, so beautiful.” Others highlighted the influence of mentors.

The theme of data science and healthcare convergence was prevalent. One respondent, drawn by the phrase “Data is the new oil,” detailed their journey: “I was captivated by the idea that data could be likened to the new oil. This comparison intrigued me, and I delved into extensive research to comprehend why data was being referred to in such a manner.” Another participant, introduced to health informatics during their undergraduate degree, shared, “Witnessing first-hand how we could leverage data and technology to enhance healthcare services and outcomes fascinated me.”

Motivations extended beyond academic and career considerations. Divine inspiration played a role for one participant who felt called to ophthalmology, stating, “I joined the field of ophthalmology as a response from a calling from God.” Family experiences also motivated individuals, with one respondent noting, “My grandparents and parents all suffer from chronic illnesses, and I want to be someone that can help my family.”

The pursuit of broader societal impact was evident in responses focused on racial equity and addressing inequalities. Participants expressed their commitment to making a positive difference, with one noting, “The potential ability to apply AI to improve health outcomes at scale is what drives me in my everyday work!”

#### Humor, Hobbies, and Advice

In exploring the quirks and unique qualities that define unique, one participant humorously recounted, “My wife didn’t allow me to carry my daughter on the stairs for four months. She was worried about me stumbling on stairs with the little one. I won’t blame her; from the time she knows me, I have fallen from stairs almost once every month.“ Another participant highlighted their expertise, stating, “I study butts for a living.” Some participants revealed unique childhood experiences, such as accidentally gluing their eyes shut or attending school with Princess Leia’s iconic hairstyle. Others showcased their diverse interests, from enjoying symphonies of nature to maintaining playlists of “embarrassing” songs on Spotify. Several participants embraced their quirks, such as one individual who humorously mentioned, “I can’t say ‘No’ to anybody who asks anything from me. If you ask me to bring a cup of coffee for you even if I have a big exam next hour, I will do that for you.” Others highlighted their enjoyment of specific activities, such as body surfing, plane spotting, and spending entire days in museums. Our questions captured a diverse range of personalities, from talkative individuals in small groups to those who describe themselves as curious but quiet. The responses collectively painted a vivid picture of the rich tapestry of quirks, passions, and unique experiences that make each participant wonderfully individual.

When asked about their hobbies and activities during spare time, participants shared a diverse range of interests and passions. One individual stated that they were deeply involved in community service, stating, “I belong to two civic organizations that do a lot for the community, especially children and mothers. I find that serving others gives me the most satisfaction.” Outdoor activities feature prominently in the responses, with participants mentioning interests in walking, snowboarding, wakeboarding, yoga, jogging, hiking, and cycling. Travel is a common theme, with individuals expressing a love for exploring new cultures and cuisines. The love for sports is evident, ranging from watching or playing football to enjoying soccer matches and participating in a 100-mile charity bike race. Artistic expressions and creative endeavors are prevalent, with participants sharing interests in photography, calligraphy, doodling, storytelling, and short film production. Family activities brought joy to some respondents. Others delve into introspective pursuits, such as writing, recording personal reflections, and contemplating the mysteries of existence. Language learning and literature appreciation emerge as shared interests, with proficiency in multiple languages and a passion for exploring original texts. Hobbies like camping, horseback riding, house cleaning, and engaging in philosophical conversations round out the vibrant spectrum of activities enjoyed during free time. The participants’ passion for various pursuits, expressed through their direct quotations, underscores the richness of their personal lives and the importance of a well-rounded approach to leisure.

We asked the participants to share the best advice they received and advice they gave to someone, and from their responses, a diverse array of wisdom emerged. Winston Churchill’s wisdom resonates with one participant, who appreciates the reminder that success is not final and failure is not fatal; it is the courage to continue that counts. The advice, “The best time to start was yesterday,” further echoes the theme of perseverance, emphasizing the importance of continuous effort and forward momentum. Such guidance aligns with the notion that “falling forward” is a key aspect of the journey toward success. One participant shared advice, “Science is not stationary or permanent. Be ready to amend your beliefs and even question your work.” Several participants emphasized the significance of relationships and personal growth. One participant underscores the importance of surrounding oneself with the right people, stating, “Surround yourself with the best people possible.” One participant shares a unique blend of academic and life advice: “Einstein only published around 4 papers; not exactly true, you get the idea.” This perspective challenges the notion of quantity over quality and encourages a thoughtful approach to knowledge creation. Another participant offered a blend of academic and life wisdom, stating, “Be open to questions and always stay curious.” This advice highlights the importance of continuous learning and curiosity’s role in academic and personal pursuits. Throughout these diverse responses, a nuanced and multifaceted approach to life emerges. Whether through personal reflections or the wisdom shared with others, these pieces of advice collectively contribute to a broader understanding of navigating challenges and pursuing growth.

**Fig. 3:**
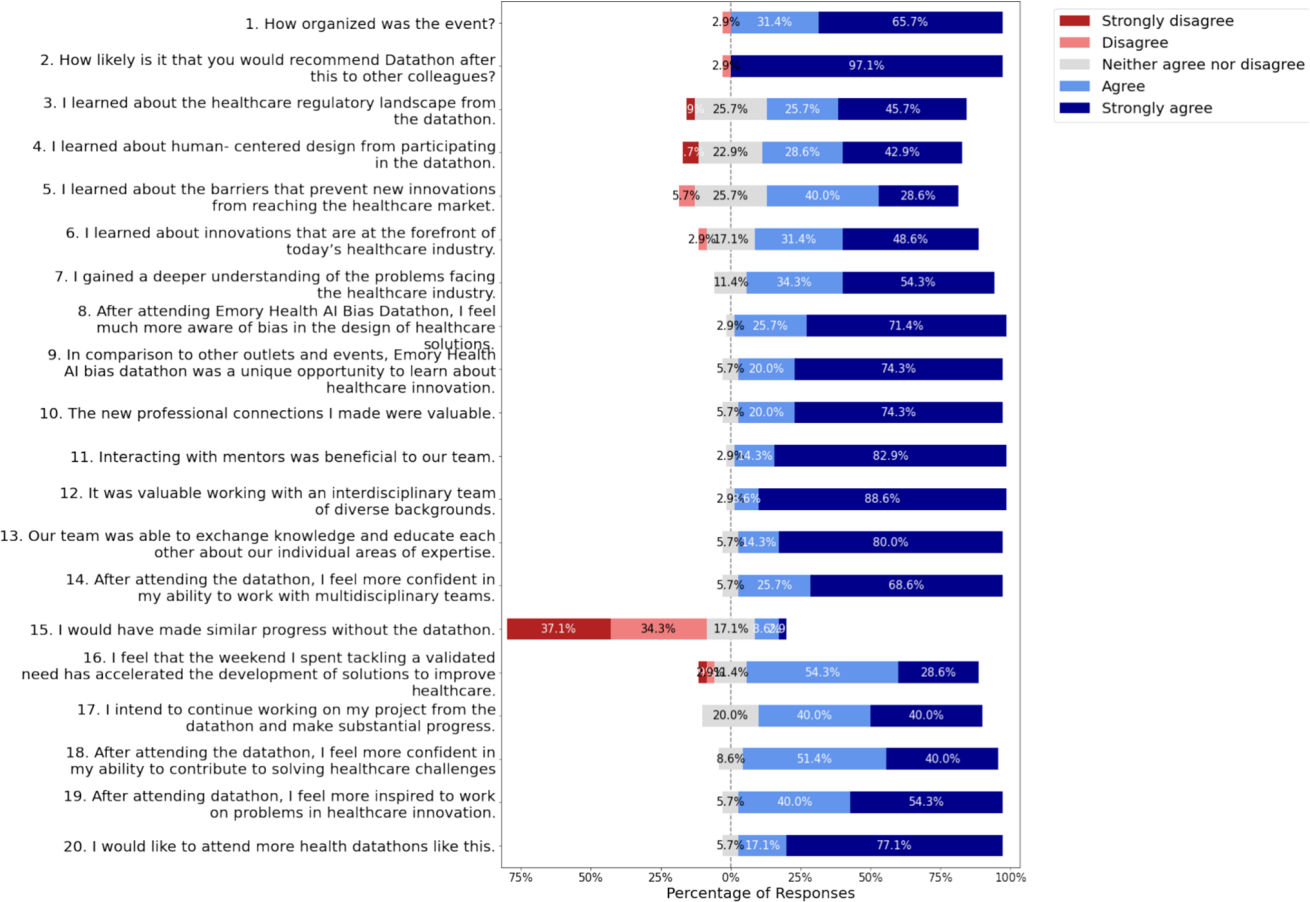
Feedback from the Participants

### Post-Event Survey

The post-event survey among 35 participants of the datathon provided interesting feedback from them. Twenty questions were asked regarding their experience and feedback for the datathon event days. Their responses to those questions are shown in a Likert chart in Fig. 3.

The responses to the aforementioned 20 questions were documented using a Likert scale ranging from 1 to 5, wherein a rating of 1 denoted a strong disagreement, and a rating of 5 signified a strong agreement. The questions posed were as follows:

1. How organized was the event?
2. How likely is it that you would recommend Datathon after this to other colleagues?
3. I learned about the healthcare regulatory landscape from the datathon.
4. I learned about human-centered design from participating in the datathon.
5. I learned about the barriers that prevent new innovations from reaching the healthcare market.
6. I learned about innovations that are at the forefront of today’s healthcare industry.
7. I gained a deeper understanding of the problems facing the healthcare industry.
8. After attending the Emory Health AI Bias Datathon, I feel much more aware of bias in the design of healthcare solutions.
9. In comparison to other outlets and events, the Emory Health AI bias datathon was a unique opportunity to learn about healthcare innovation.
10. The new professional connections I made were valuable.
11. Interacting with mentors was beneficial to our team.
12. It was valuable working with an interdisciplinary team of diverse backgrounds.
13. Our team was able to exchange knowledge and educate each other about our individual areas of expertise.
14. After attending the datathon, I feel more confident in my ability to work with multidisciplinary teams.
15. I would have made similar progress without the datathon.
16. I feel that the weekend I spent tackling a validated need has accelerated the development of solutions to improve healthcare.
17. I intend to continue working on my project from the datathon and make substantial progress.
18. After attending the datathon, I feel more confident in my ability to contribute to solving healthcare challenges
19. After attending the datathon, I feel more inspired to work on problems in healthcare innovation.
20. I would like to attend more health datathons like this.

A notable 77.14% of participants expressed a strong desire to attend more health datathons, indicating a keen interest in future events of a similar nature. Post-datathon, a significant 54.29% strongly felt more inspired to tackle problems in healthcare innovation, with an additional 40.00% in agreement. Confidence levels surged, as 51.43% strongly believed in their ability to contribute to solving healthcare challenges, complemented by 40.00% in agreement. A noteworthy 54.29% felt that the datathon weekend, dedicated to addressing validated needs, accelerated the development of healthcare solutions. Collaboration and knowledge exchange within teams were highly valued, with 80.00% strongly agreeing that their team effectively exchanged expertise. Working with interdisciplinary teams garnered substantial appreciation, with 88.57% finding it valuable and 68.57% feeling more confident in their ability to work with such teams after the datathon. Participants commended the event’s organization, as 65.71% strongly agreed that it was well-organized. Impressively, a resounding 97.14% expressed a strong likelihood of recommending the datathon to their colleagues. In essence, the responses reflect a highly positive and enriching experience, highlighting the datathon’s success in fostering collaboration, knowledge acquisition, and inspiration within the realm of healthcare innovation.

## Discussion

One of the interesting facts we observed during the organization of the datathon event is that the participants who registered for the events provided interesting facts about themselves or their experience during the registration. The responses from the participants in the datathon, captured through a Likert scale ranging from 1 to 5, reveal a highly positive and engaging experience. A significant majority expressed a keen interest in attending more health datathons, emphasizing the value they found in the event. Participants overwhelmingly reported feeling inspired and confident in addressing healthcare innovation challenges post-datathon. Notably, the interdisciplinary nature of the teams, coupled with mentor interactions, proved highly beneficial, fostering knowledge exchange and team education. The unique learning opportunities presented by the datathon, particularly in healthcare innovation, human-centered design, and understanding regulatory landscapes, were well-received. Furthermore, the event significantly heightened awareness of bias in healthcare solutions. The overwhelmingly positive responses regarding team collaboration, knowledge exchange, and newfound confidence collectively underscore the success of the datathon in achieving its educational and collaborative objectives. The high likelihood of participants recommending the datathon to their colleagues reflects the overall satisfaction and perceived value derived from the event. Overall, these insights affirm the datathon’s effectiveness in fostering innovation, interdisciplinary collaboration, and knowledge enrichment within the healthcare domain.

### Lessons Learned

The datathon teams had a number of mentors assigned exclusively for each team from various backgrounds and specialties. The profession distribution among the mentors is shown in Fig. 2. Along with that, the organizers and the senior members of Emory University were the judges for the datathon. From this experience of the datathon, we have learnt that participants of the datathon come from various background. Thus, while it is expected from them to have a bit of prior knowledge in health and artificial intelligence, basic programming, the organizers plan to introduce summer school before datathon event for participant for the next annual datathons in Emory University. To bear the expense of summer school and corresponding cost of attendees of it, grant proposals preparation, and submissions are going on. Amazon Web Services (AWS) has assisted significantly on providing a proper platform to host the required infrastructure, i.e., server for the datathon. Thus, for the future programs, organizers will continue using AWS service.

## Conclusion

The findings from this experience report from the Emory Health AI Bias Datathon held in August 2023 highlight the event’s impact on participants, emphasizing their resilience, dedication, and innovative contributions. The breakdown of participant demographics, responses to post-event surveys, and backgrounds provide a comprehensive understanding of the diverse cohort involved. The positive responses from the participants regarding the organization, learning opportunities, and collaborative experiences affirm the datathon’s effectiveness in providing a platform for meaningful interactions, knowledge exchange, and skill development. We can conclude that the Emory Health AI Bias Datathon serves as a model for future events, highlighting the importance of fostering diversity, collaboration, and innovation in addressing complex challenges within the realm of health data science. The lessons learned and experiences gained during this datathon will undoubtedly contribute to the continued success and improvement of future events in this rapidly evolving field.

## References

[1] Little, C.: Undergraduate research as a student engagement springboard: Exploring the longer-term reported benefits of participation in a research conference. Educational Research. **62**(2), 229–245 (2020)

[2] Anslow, C., Brosz, J., Maurer, F., Boyes, M.: Datathons: an experience report of data hackathons for data science education. In: Proceedings of the 47th ACM Technical Symposium on Computing Science Education, pp. 615–620 (2016)

[3] Longo, A., Zappatore, M., Martella, A., Rucco, C.: Enhancing data education with datathons: an experience with open data on renewable energy systems. In: 1st International Workshop on Data Systems Education, pp. 26–31 (2022)

[4] Wang, J.K., Roy, S.K., Barry, M., Chang, R.T., Bhatt, A.S.: Institutionalizing healthcare hackathons to promote diversity in collaboration in medicine. BMC medical education. **18**(1), 1–9 (2018) 30458759 10.1186/s12909-018-1385-x PMC6245929

[5] Fritz, S., Milligan, I., Ruest, N., Lin, J.: Fostering community engagement through datathon events: The archives unleashed experience. (2021)

[6] Tan, S.C., Evans, T., Hensman, T., Durie, M., Secombe, P., Pilcher, D.: Clinical informatics needs to be a competency for intensive care training. Critical Care and Resuscitation. **25**(1), 6–8 (2023) 37876988 10.1016/j.ccrj.2023.04.003 PMC10581266

[7] Schiff, D., Borenstein, J., Biddle, J., Laas, K.: Ai ethics in the public, private, and ngo sectors: A review of a global document collection. IEEE Transactions on Technology and Society. **2**(1), 31–42 (2021)

[8] Purkayastha, S., Price, A., Biswas, R., Ganesh, A.J., Otero, P.: From dyadic ties to information infrastructures: care-coordination between patients, providers, students and researchers. Yearbook of medical informatics. **24**(01), 68–74 (2015) 10.15265/IY-2015-008 PMC4587042 26123908

[9] Cosgriff, C.V., Charpignon, M., Moukheiber, D., Lough, M.E., Gichoya, J., Stone, D.J., Celi, L.A.: Village mentoring and hive learning: The mit critical data experience. Iscience. **24**(6) (2021) 10.1016/j.isci.2021.102656 PMC8209268 34169236

[10] Peleg, M., Reichman, A., Shachar, S., Gadot, T., Avgil Tsadok, M., Azaria, M., Dunkelman, O., Hassid, S., Partem, D., Shmailov, M., *et al.*: Collaboration between government and research community to respond to covid-19: Israel’s case. Journal of Open Innovation: Technology, Market, and Complexity. **7**(4), 208 (2021)

[11] Boom, W., Hoy, M., Sankaran, J., Liu, M., Chahed, H., Feng, M., See, K.C.: The search for optimal oxygen saturation targets in critically ill patients: observational data from large icu databases. Chest. **157**(3), 566–573 (2020) 31589844 10.1016/j.chest.2019.09.015

[12] Sellergren, A.B., Chen, C., Nabulsi, Z., Li, Y., Maschinot, A., Sarna, A., Huang, J., Lau, C., Kalidindi, S.R., Etemadi, M., *et al.*: Simplified transfer learning for chest radiography models using less data. Radiology. **305**(2), 454–465 (2022) 35852426 10.1148/radiol.212482

[13] Nicholas, I., Kuo, H., Perez-Concha, O., Hanly, M., Mnatzaganian, E., Hao, B., Di Sipio, M., Yu, G., Vanjara, J., Valerie, I.C., *et al.*: Enriching data science and health care education: Application and impact of synthetic data sets through the health gym project. JMIR Medical Education. **10**(1), 51388 (2024) 10.2196/51388 PMC10828942 38227356

[14] Paris, N., Lamer, A., Parrot, A.: Transformation and evaluation of the mimic database in the omop common data model: development and usability study. JMIR Medical Informatics. **9**(12), 30970 (2021) 10.2196/30970 PMC8715361 34904958

[15] Li, P., Xie, C., Pollard, T., Johnson, A.E.W., Cao, D., Kang, H., Liang, H., Zhang, Y., Liu, X., Fan, Y., *et al.*: Promoting secondary analysis of electronic medical records in china: summary of the plagh-mit critical data conference and health datathon. JMIR Medical Informatics. **5**(4), 7380 (2017) 10.2196/medinform.7380 PMC5705855 29138126

[16] Serpa Neto, A., Kugener, G., Bulgarelli, L., Rabello Filho, R., Hoz, M.Á.A.d.l., Johnson, A.E., Paik, K.E., Torres, F., Xie, C., Amaro Júnior, E., *et al.*: First brazilian datathon in critical care. Revista Brasileira de terapia intensiva. **30**, 6–8 (2018) 29742215 10.5935/0103-507X.20180006 PMC5885224

[17] Toledo Piza, F.M., Celi, L.A., Deliberato, R.O., Bulgarelli, L., Carvalho, F.R.T., Rabello Filho, R., La Hoz, M.A.A., Kesselheim, J.C.: Assessing team effectiveness and affective learning in a datathon. International journal of medical informatics. **112**, 40–44 (2018) 10.1016/j.ijmedinf.2018.01.005 29500020

[18] Lyndon, M.P., Pathanasethpong, A., Henning, M.A., Chen, Y., Celi, L.A.: Measuring the learning outcomes of datathons. BMJ Innovations. **8**(2) (2022)

[19] Celi, L.A., Davidzon, G., Johnson, A.E., Komorowski, M., Marshall, D.C., Nair, S.S., Phillips, C.T., Pollard, T.J., Raffa, J.D., Salciccioli, J.D., *et al.*: Bridging the health data divide. Journal of medical Internet research. **18**(12), 325 (2016) 10.2196/jmir.6400 PMC5209608 27998877

[20] Sobel, J., Almog, R., Celi, L., Yablowitz, M., Eytan, D., Behar, J.: How to organise a datathon for bridging between data science and healthcare? insights from the technion-rambam machine learning in healthcare datathon event. BMJ Health & Care Informatics. **30**(1) (2023) 10.1136/bmjhci-2023-100736 PMC10496710 37696642

[21] Aboab, J., Celi, L.A., Charlton, P., Feng, M., Ghassemi, M., Marshall, D.C., Mayaud, L., Naumann, T., McCague, N., Paik, K.E., *et al.*: A “datathon” model to support cross-disciplinary collaboration. Science Translational Medicine. **8**(333), 333–83338 (2016) 10.1126/scitranslmed.aad9072 PMC5679209 27053770

[22] Jeong, J.J., Vey, B.L., Bhimireddy, A., Kim, T., Santos, T., Correa, R., Dutt, R., Mosunjac, M., Oprea-Ilies, G., Smith, G., *et al.*: The emory breast imaging dataset (embed): A racially diverse, granular dataset of 3.4 million screening and diagnostic mammographic images. Radiology: Artificial Intelligence. **5**(1), 220047 (2023) 10.1148/ryai.220047 PMC9885379 36721407

